# Supplementary material for: De novo-designed transmembrane domains tune engineered receptor functions
Source: eLife. 2022 May 4;11:e75660. doi: 10.7554/eLife.75660 (PMC9068223; doi:10.7554/eLife.75660)
Supplement: Supplementary file 1. [file elife-75660-supp1.docx]

**RosettaScripts and commandlines for “*De novo* designed transmembrane domains tune engineered receptor functions”**

Assaf Elazar, Nicholas J. Chandler, Ashleigh S. Davey, Jonathan Y. Weinstein, Julie V. Nguyen, Raphael Trenker, Ryan S. Cross, Misty R. Jenkins, Melissa J. Call, Matthew E. Call and Sarel J. Fleishman

**Fold, Dock, and Design**

<ROSETTASCRIPTS>

<SCOREFXNS>

<ScoreFunction name="score0" weights="%%score_func_0%%" symmetric="1">

<Reweight scoretype="mp_helicality" weight="100"/>

</ScoreFunction>

<ScoreFunction name="score1" weights="%%score_func_1%%" symmetric="1">

<Reweight scoretype="mp_helicality" weight="100"/>

</ScoreFunction>

<ScoreFunction name="score2" weights="%%score_func_2%%" symmetric="1">

<Reweight scoretype="mp_helicality" weight="100"/>

</ScoreFunction>

<ScoreFunction name="score3" weights="%%score_func_3%%" symmetric="1">

<Reweight scoretype="mp_helicality" weight="100"/>

</ScoreFunction>

<ScoreFunction name="score5" weights="%%score_func_5%%" symmetric="1">

<Reweight scoretype="mp_helicality" weight="100"/>

</ScoreFunction>

<ScoreFunction name="beta" weights="ref2015_memb" symmetric="1">

<Reweight scoretype="mp_helicality" weight="100"/>

</ScoreFunction>

<ScoreFunction name="betaNotSymm" weights="ref2015_memb" symmetric="0">

<Reweight scoretype="mp_helicality" weight="100"/>

</ScoreFunction>

<ScoreFunction name="helicality" symmetric="1">

<Reweight scoretype="mp_helicality" weight="1"/>

</ScoreFunction>

</SCOREFXNS>

<TASKOPERATIONS>

<InitializeFromCommandline name="init"/>

<RestrictToRepacking name="rtr"/>

</TASKOPERATIONS>

<MOVERS>

<PyMOLMover name="pmm" keep_history="1"/>

<SetupForSymmetry name="symm" definition="%%symm_file%%"/>

<SymmetricAddMembraneMover name="add_memb" membrane_core="%%membrane_core%%" steepness="%%steepness%%">

<Span start="%%span_start_1%%" end="%%span_end_1%%" orientation="%%span_orientation_1%%"/>

<Span start="%%span_start_2%%" end="%%span_end_2%%" orientation="%%span_orientation_2%%"/>

</SymmetricAddMembraneMover>

<MembranePositionFromTopologyMover name="init_pos"/>

<FastRelax name="fast_relax" scorefxn="beta"/>

Fragment movers

<SingleFragmentMover name="frag9" fragments="%%frags9mers%%" policy="uniform"/>

<SingleFragmentMover name="frag3" fragments="%%frags3mers%%" policy="smooth"/>

Fold-and-dock specific movers

<SymFoldandDockRbTrialMover name="rbtrial" rot_mag="8.0" trans_mag="3.0" rotate_anchor_to_x="1"/>

<SymFoldandDockRbTrialMover name="rbtrial_smooth" rot_mag="1.0" trans_mag="0.1" rotate_anchor_to_x="1"/>

<SymFoldandDockMoveRbJumpMover name="rbjump"/>

<SymFoldandDockSlideTrialMover name="slidetrial"/>

Random movers

<RandomMover name="early_stage_moveset" movers="frag9,rbtrial,rbjump,slidetrial" weights="1.0,0.2,1.0,0.1" repeats="1"/>

<RandomMover name="final_stage_moveset" movers="frag3,rbtrial_smooth,rbjump,slidetrial" weights="1.0,0.2,1.0,0.1" repeats="1"/>

Monte Carlo Movers

<GenericMonteCarlo name="stage1" scorefxn_name="score0" mover_name="early_stage_moveset" temperature="2.0" trials="200" recover_low="1"/>

<GenericMonteCarlo name="stage2" scorefxn_name="score1" mover_name="early_stage_moveset" temperature="2.0" trials="200" recover_low="1"/>

<GenericMonteCarlo name="stage3a" scorefxn_name="score2" mover_name="early_stage_moveset" temperature="2.0" trials="20" recover_low="1"/>

<GenericMonteCarlo name="stage3b" scorefxn_name="score5" mover_name="early_stage_moveset" temperature="2.0" trials="20" recover_low="1"/>

<GenericMonteCarlo name="stage4" scorefxn_name="score3" mover_name="final_stage_moveset" temperature="2.0" trials="400" recover_low="1"/>

Special stage 3 logic

<ParsedProtocol name="stage3_cyc">

<Add mover="stage3a"/>

<Add mover="stage3b"/>

</ParsedProtocol>

<LoopOver name="stage3" mover_name="stage3_cyc" iterations="5" drift="1"/>

Converts the centroid-level pose to fullatom for scoring

<SwitchResidueTypeSetMover name="fullatom" set="fa_standard"/>

<ExtractAsymmetricPose name="extract_asp"/>

<!--<SymPackRotamersMover name="soft_design" scorefxn="beta_soft" task_operations="init"/>-->

<TaskAwareSymMinMover name="hard_min" scorefxn="beta" chi="1" bb="1" rb="1" task_operations="init"/>

<SymPackRotamersMover name="hard_design" scorefxn="beta" task_operations="init"/>

<RotamerTrialsMinMover name="RTmin" scorefxn="beta" task_operations="init,rtr"/>

</MOVERS>

<FILTERS>

<ScoreType name="total" scorefxn="beta" score_type="total_score" confidence="0" threshold="0"/>

<Sasa name="a_sasa" confidence="0"/>

<ResidueLipophilicity name="a_res_solv" threshold="1000" confidence="0"/>

<SpanTopologyMatchPose name="a_span_topo" confidence="0"/>

<Ddg name="a_ddg" scorefxn="betaNotSymm" chain_num="2" repeats="5" extreme_value_removal="true" confidence="0"/>

<PackStat name="a_pack" confidence="0"/>

<BuriedUnsatHbonds2 name="a_unsat" scorefxn="beta" confidence="0"/>

<ShapeComplementarity name="a_shape" confidence="0"/>

<TMsSpanMembrane name="a_tms_span" confidence="1"/>

<TMsSpanMembrane name="a_tms_span_fa" confidence="1" min_distance="25"/>

<HelixHelixAngle name="a_hha_ang" angle_or_dist="angle" start_helix_1="%%span_start_1%%" end_helix_1="%%span_end_1%%" start_helix_2="%%span_start_2%%" end_helix_2="%%span_end_2%%" confidence="0"/>

<HelixHelixAngle name="a_hha_dst_vec" angle_or_dist="dist" dist_by_atom="0" start_helix_1="%%span_start_1%%" end_helix_1="%%span_end_1%%" start_helix_2="%%span_start_2%%" end_helix_2="%%span_end_2%%" confidence="0"/>

<HelixHelixAngle name="a_hha_dst_atm" angle_or_dist="dist" dist_by_atom="1" start_helix_1="%%span_start_1%%" end_helix_1="%%span_end_1%%" start_helix_2="%%span_start_2%%" end_helix_2="%%span_end_2%%" confidence="0"/>

<ScoreType name="a_helicality" scorefxn="helicality" score_type="total_score" confidence="1" threshold="3"/>

<TMsAAComp name="a_tms_aa_comp" confidence="0" threshold="0"/>

</FILTERS>

<PROTOCOLS>

<Add mover="symm"/>

<Add mover="add_memb"/>

<Add mover="stage1"/>

<Add mover="stage2"/>

<Add mover="stage3"/>

<Add mover="stage4"/>

<Add filter="a_helicality"/>

<Add mover="fullatom"/>

<Add filter="a_tms_span_fa"/>

!!!! design !!!!

<Add mover="hard_design"/>

<Add mover="hard_min"/>

<Add mover="hard_design"/>

<Add mover="hard_min"/>

<Add mover="RTmin"/>

<Add mover="RTmin"/>

<Add filter="a_tms_span"/>

<Add mover="fast_relax"/>

<Add mover="pmm"/> # DO I WANT THIS HERE?

<Add filter="total"/>

<Add filter="a_sasa"/>

<Add filter="a_res_solv"/>

<Add filter="a_span_topo"/>

<Add filter="a_helicality"/>

# important to remove Symmetry so RMSD can be calculated

<Add mover="extract_asp"/>

<Add filter="a_res_solv"/>

<Add filter="a_pack"/>

<Add filter="a_unsat"/>

<Add filter="a_shape"/>

<Add filter="a_ddg"/>

<Add filter="a_hha_ang"/>

<Add filter="a_hha_dst_vec"/>

<Add filter="a_hha_dst_atm"/>

<Add filter="a_tms_aa_comp"/>

</PROTOCOLS>

<OUTPUT scorefxn="betaNotSymm"/>

</ROSETTASCRIPTS>

**Command line**

~/Rosetta/main/source/bin/rosetta_scripts.default.linuxgccreleas -database Rosetta/main/database @**flags_fdd**

flags_fdd:

# general data

-parser:protocol fold_dock_design.xml

-in:file:fasta ploy_V.fasta

-in:file:native 24.pdb

-overwrite

-use_input_sc

-nstruct 12

-jd2:ntrials 10

#-mute all

# fragment stuff

-parser:script_vars frags9mers=frags_9ploy_V_pdbtm.200.9mers

-parser:script_vars frags3mers=frags_3ploy_V_pdbtm.200.3mers

-parser:script_vars symm_file=C2.symm

# membrane spans:

-parser:script_vars span_starts=1A

-parser:script_vars span_ends=24A

-parser:script_vars span_oris=out2in

-parser:script_vars span_start_1=1

-parser:script_vars span_end_1=24

-parser:script_vars span_orientation_1=out2in

-parser:script_vars span_start_2=25

-parser:script_vars span_end_2=48

-parser:script_vars span_orientation_2=out2in

-parser:script_vars span_starts=1A

-parser:script_vars span_ends=24A

-parser:script_vars span_oris=out2in

-parser:script_vars score_func_0=score0_memb

-parser:script_vars score_func_1=score1_memb

-parser:script_vars score_func_2=score2_memb

-parser:script_vars score_func_3=score3_memb

-parser:script_vars score_func_5=score5_memb

# energy function stuff:

-parser:script_vars energy_function=ref

# membrane adjustments

-mp:scoring:hbond

-parser:script_vars steepness=4

-parser:script_vars membrane_core=10

**Sequence diversification**

<ROSETTASCRIPTS>

<SCOREFXNS>

<ScoreFunction name="ref" weights="ref2015_memb" symmetric="1">

<Reweight scoretype="fa_mpenv_smooth" weight="0.000001"/>

<Reweight scoretype="coordinate_constraint" weight="0.4"/>

</ScoreFunction>

<ScoreFunction name="ref_no_cst" weights="ref2015_memb" symmetric="1">

<Reweight scoretype="fa_mpenv_smooth" weight="0.000001"/>

</ScoreFunction>

<ScoreFunction name="helicality" symmetric="1">

<Reweight scoretype="mp_helicality" weight="1"/>

</ScoreFunction>

</SCOREFXNS>

<RESIDUE_SELECTORS>

<ResidueName name="gly" residue_name3="GLY"/>

<ResidueName name="asn_gln" residue_name3="ASN,GLN"/>

<Index name="all_aas" resnums="1-48"/>

<Not name="not_aas" selector="all_aas"/>

</RESIDUE_SELECTORS>

<TASKOPERATIONS>

<OperateOnResidueSubset name="keep_gly" selector="gly">

<RestrictToRepackingRLT/>

</OperateOnResidueSubset>

<OperateOnResidueSubset name="keep_qn" selector="asn_gln">

<RestrictToRepackingRLT/>

</OperateOnResidueSubset>

<OperateOnResidueSubset name="freeze_not_aas" selector="not_aas">

<PreventRepackingRLT/>

</OperateOnResidueSubset>

<RestrictToRepacking name="rtr"/>

<RestrictAbsentCanonicalAAS name="to_restrict_avlimfwyst" resnum="0" keep_aas="GAVIFSTYW"/>

<InitializeFromCommandline name="init"/>

</TASKOPERATIONS>

<FILTERS>

<ResidueLipophilicity name="a_res_lipo" confidence="0"/>

<ScoreType name="a_total" scorefxn="ref" score_type="total_score" confidence="0" threshold="0"/>

<ScoreType name="a_total_final" scorefxn="ref" score_type="total_score" confidence="1" threshold="-140"/>

<Sasa name="a_sasa" confidence="0"/>

<SpanTopologyMatchPose name="a_span_topo" confidence="0"/>

<Ddg name="a_ddg" scorefxn="ref_no_cst" confidence="0" />

<PackStat name="a_pack" confidence="1" threshold="0.3"/>

<BuriedUnsatHbonds2 name="a_unsat" scorefxn="ref" confidence="0"/>

<ShapeComplementarity name="a_shape" confidence="0"/>

<TMsSpanMembrane name="a_tms_span" confidence="0"/>

<TMsSpanMembrane name="a_tms_span_fa" confidence="0" min_distance="25"/>

<HelixHelixAngle name="a_hha_ang" angle_or_dist="angle" start_helix_1="%%s1%%" end_helix_1="%%e1%%" start_helix_2="%%s2%%" end_helix_2="%%e2%%" confidence="0"/>

<MembAccesResidueLipophilicity name="a_marl" confidence="0" verbose="0"/>

<TMsAAComp name="a_tms_aa_comp" confidence="0" threshold="0"/>

<BindingStrain name="a_bind" scorefxn="ref_no_cst" jump="1" confidence="1" threshold="5"/>

<ScoreType name="a_helicality" scorefxn="helicality" score_type="total_score" confidence="1" threshold="3"/>

<Sigmoid name="a_total_sig" filter="a_total" steepness="0.5" offset="20" negate="0" confidence="0"/>

<Sigmoid name="a_ddg_sig_10" filter="a_ddg" steepness="1" offset="10" negate="0" confidence="0"/>

<Sigmoid name="a_ddg_sig_2" filter="a_ddg" steepness="1" offset="3" negate="0" confidence="0"/>

<Sigmoid name="a_comp_sig" filter="a_tms_aa_comp" steepness="50" offset="0.05" negate="0" confidence="0"/>

<Operator name="a_obj_func_ddg_10" filters="a_total_sig,a_ddg_sig_10,a_comp_sig" operation="PRODUCT" logarithm="1" threshold="100000" confidence="0" negate="1"/>

<Operator name="a_obj_func_ddg_2" filters="a_total_sig,a_ddg_sig_2,a_comp_sig" operation="PRODUCT" logarithm="1" threshold="100000" confidence="0" negate="1"/>

</FILTERS>

<MOVERS>

<SymmetricAddMembraneMover name="add_memb" membrane_core="%%memb_core%%" steepness="%%steepness%%">

<Span start="%%s1%%" end="%%e1%%" orientation="%%o1%%"/>

<Span start="%%s2%%" end="%%e2%%" orientation="%%o2%%"/>

</SymmetricAddMembraneMover>

<RandomMutation name="mutate" task_operations="freeze_not_aas,init,to_restrict_avlimfwyst,keep_gly,keep_qn" scorefxn="ref"/>

<VirtualRoot name="virt_root"/>

<AtomCoordinateCstMover name="atom_cst" coord_dev="0.5" bounded="false" native="false"/>

<TaskAwareSymMinMover name="min_mover" scorefxn="ref" chi="1" bb="1" rb="1" task_operations="init,rtr"/>

<ParsedProtocol name="mutate_min">

<Add mover="mutate"/>

<Add mover="min_mover"/>

</ParsedProtocol>

<GenericMonteCarlo name="gmc_sigs_1" filter_name="a_obj_func_ddg_10" preapply="0" mover_name="mutate_min" temperature="0.1" trials="240" recover_low="1" reset_baselines="1"/>

<GenericMonteCarlo name="gmc_sigs_2" filter_name="a_obj_func_ddg_2" preapply="0" mover_name="mutate_min" temperature="0.1" trials="80" recover_low="1" reset_baselines="1"/>

<GenericSimulatedAnnealer name="sim_anneal" mover_name="mutate_min" filter_name="a_obj_func_ddg_2" trials="120" sample_type="low" recover_low="1" preapply="0" reset_baselines="1" history="10"/>

<SetupForSymmetry name="symm" definition="%%symm_file%%" preserve_datacache="false"/>

</MOVERS>

<PROTOCOLS>

<Add mover="symm"/>

<Add mover="add_memb"/>

<Add mover="atom_cst"/>

<Add mover="min_mover"/>

<Add mover="sim_anneal"/>

<Add mover="min_mover"/>

<Add filter="a_total_sig"/>

<Add filter="a_total_final"/>

<Add filter="a_ddg_sig_2"/>

<Add filter="a_ddg_sig_10"/>

<Add filter="a_comp_sig"/>

<Add filter="a_obj_func_ddg_2"/>

<Add filter="a_obj_func_ddg_10"/>

<Add filter="a_tms_aa_comp"/>

<Add filter="a_res_lipo"/>

<Add filter="a_marl"/>

<Add filter="a_sasa"/>

<Add filter="a_total"/>

<Add filter="a_ddg"/>

<Add filter="a_bind"/>

<Add filter="a_pack"/>

<Add filter="a_unsat"/>

<Add filter="a_shape"/>

<Add filter="a_tms_span"/>

<Add filter="a_helicality"/>

<Add filter="a_tms_aa_comp"/>

</PROTOCOLS>

<OUTPUT scorefxn="ref"/>

</ROSETTASCRIPTS>

**Command line**

~/Rosetta/main/source/bin/rosetta_scripts.default.linuxgccreleas -database Rosetta/main/database @**flags_seq_divers**

flags_seq_divers:

-parser:protocol GMC_seq_diversifier.xml

-overwrite

-parser:script_vars memb_core=10

-parser:script_vars steepness=4

-mute all

-nstruct 10

-parser:script_vars s1=1

-parser:script_vars s2=24

-parser:script_vars e1=25

-parser:script_vars e2=48

-parser:script_vars o1=out2in

-parser:script_vars o2=out2in

-mp:scoring:hbond

-jd2:ntrials 1000000

-use_input_sc

-s seed_INPUT.pdb

-parser:script_vars symm_file=seed.symm

**FilterScan**

<ROSETTASCRIPTS>

<SCOREFXNS>

<ScoreFunction name="ddg_sfx" weights="%%scorefxn%%" symmetric="1"/>

<ScoreFunction name="full" weights="ref2015_memb" symmetric="1">

<Reweight scoretype="coordinate_constraint" weight="%%cst_value%%"/>

</ScoreFunction>

<ScoreFunction name="soft" weights="ref2015_soft" symmetric="1">

<Reweight scoretype="mp_res_lipo" weight="1"/>

<Reweight scoretype="coordinate_constraint" weight="%%cst_value%%"/>

</ScoreFunction>

</SCOREFXNS>

<TASKOPERATIONS>

<InitializeFromCommandline name="init"/>

<RestrictToRepacking name="rtr"/>

<DesignAround name="des_around" design_shell="0.1" resnums="%%current_res%%" repack_shell="8.0"/>

<OperateOnResidueSubset name="restrict_res">

<Index resnums="%%res_to_restrict%%"/>

<RestrictToRepackingRLT/>

</OperateOnResidueSubset>

<OperateOnResidueSubset name="fix_res">

<Index resnums="%%res_to_fix%%"/>

<PreventRepackingRLT/>

</OperateOnResidueSubset>

</TASKOPERATIONS>

<MOVERS>

<SetupForSymmetry name="symm" definition="%%symm_file%%"/>

<SymmetricAddMembraneMover name="add_memb" membrane_core="10" steepness="4">

<Span start="1" end="24" orientation="in2out"/>

<Span start="25" end="48" orientation="in2out"/>

</SymmetricAddMembraneMover>

<TransformIntoMembraneMover name="tramsform" />

<AtomCoordinateCstMover name="atom_cst" coord_dev="0.5" bounded="false" native="false"/>

<MinMover name="min_all" scorefxn="ddg_sfx" chi="1" bb="1" jump="%%jump%%"/>#scorefxn_full

<SymPackRotamersMover name="soft_repack" scorefxn="soft" task_operations="init,rtr"/>

<SymPackRotamersMover name="hard_repack" scorefxn="full" task_operations="init,rtr"/>

<SymRotamerTrialsMover name="RTmin" scorefxn="full" task_operations="init,rtr"/>

<SymMinMover name="soft_min" scorefxn="soft" chi="1" bb="1" jump="0"/>

<SymMinMover name="hard_min" scorefxn="full" chi="1" bb="1" jump="0"/>

<ParsedProtocol name="refinement_block"> #10 movers

<Add mover_name="soft_repack"/>

<Add mover_name="soft_min"/>

<Add mover_name="soft_repack"/>

<Add mover_name="hard_min"/>

<Add mover_name="hard_repack"/>

<Add mover_name="hard_min"/>

<Add mover_name="hard_repack"/>

Add mover_name="RTmin"/>

Add mover_name="RTmin"/>

<Add mover_name="hard_min"/>

</ParsedProtocol>

<LoopOver name="iter4" mover_name="refinement_block" iterations="4"/>

</MOVERS>

<FILTERS>

<Ddg name="ddg" scorefxn="ddg_sfx" threshold="0" repeats="5"/>#chain_num="2"

<ScoreType name="stability_score_full" scorefxn="full" score_type="total_score" threshold="0.0"/>

<Delta name="delta_score_full" filter="stability_score_full" upper="1" lower="0" range="0.5"/> #upper and lower are booleans. Delta filters out all the mutations that are worse or better by less than -0.55R.E.U

Delta name="delta_score_full" filter="stability_score_full" upper="1" lower="0" range="0.5"/> #upper and lower are booleans. Delta filters out all the mutations that are worse or better by less than -0.55R.E.U

FilterScan name="filter_scan" scorefxn="ddg_sfx" relax_mover="min_all" keep_native="%%keep_n%%" task_operations="init,des_around,fix_res,restrict_res" delta_filters="delta_score_full" delta="true" resfile_name="%%resfiles_path%%/res_%%current_res%%" report_all="1" delta_filter_thresholds="%%fs_thresholds%%" score_log_file="%%scores_path%%/res%%current_res%%_score_full.log" dump_pdb="1" />

<FilterScan name="filter_scan" scorefxn="full" relax_mover="iter4" keep_native="%%keep_n%%" task_operations="init,des_around,fix_res,restrict_res" delta_filters="delta_score_full" delta="true" resfile_name="%%resfiles_path%%/res_%%current_res%%" report_all="1" delta_filter_thresholds="%%fs_thresholds%%" score_log_file="%%scores_path%%/res%%current_res%%_score_full.log" dump_pdb="0" />

</FILTERS>

<PROTOCOLS>

<Add mover="symm"/>

<Add mover="add_memb"/>

Add mover="tramsform"/>

<Add mover="atom_cst"/>

<Add filter="filter_scan"/>

</PROTOCOLS>

</ROSETTASCRIPTS>

**Command line**

~/Rosetta/main/source/bin/rosetta_scripts.default.linuxgccreleas -database Rosetta/main/database @**flags_filterscan**

Flags_filterscan:

# general data

-parser:protocol filterscan_auto_refine_SYMM.xml

#-database #path to database

-overwrite

# membrane spans: #changed to in2out 23Feb17 for experimental reasons

-parser:script_vars res_to_fix=1A

-parser:script_vars res_to_restrict=1A

-parser:script_vars cst_value=0.4

-parser:script_vars jump=1

-parser:script_vars scorefxn=ref2015_memb

-parser:script_vars span_orientation_2=in2out

-parser:script_vars fs_thresholds=0.0,0.5,1.0,1.5,2.0,2.5,3.0,3.5,4.0,4.5,5.0,5.5,6.0,6.5,7.0,8.0,9.0,10.0,11.0,12.0

-parser:script_vars keep_n=1

#-score::elec_memb_sig_die

#-corrections::beta_nov16

#-score:memb_fa_sol

-mp:scoring:hbond

-use_input_sc

-parser:script_vars current_res=3

-parser:script_vars pdb_dump=#filterscan/pdbs/3_

-out:path:score score

-out:path:pdb pdbs

-parser:script_vars resfiles_path=./

-parser:script_vars scores_path=./

-parser:script_vars symm_file=seed.symm

***ab-initio* structure prediction**

<ROSETTASCRIPTS>

<TASKOPERATIONS>

<InitializeFromCommandline name="init"/>

<RestrictToRepacking name="rtr"/>

</TASKOPERATIONS>

<SCOREFXNS>

<ScoreFunction name="score0" weights="%%score_func_0%%" symmetric="1">

<Reweight scoretype="mp_helicality" weight="0"/>

<Reweight scoretype="mp_span_ang" weight="0"/>

</ScoreFunction>

<ScoreFunction name="score1" weights="%%score_func_1%%" symmetric="1">

<Reweight scoretype="mp_helicality" weight="0"/>

<Reweight scoretype="mp_span_ang" weight="0"/>

<Reweight scoretype="mp_nonhelix" weight="0"/>

</ScoreFunction>

<ScoreFunction name="score2" weights="%%score_func_2%%" symmetric="1">

<Reweight scoretype="mp_helicality" weight="0"/>

<Reweight scoretype="mp_span_ang" weight="0"/>

<Reweight scoretype="mp_nonhelix" weight="0"/>

</ScoreFunction>

<ScoreFunction name="score3" weights="%%score_func_3%%" symmetric="1">

<Reweight scoretype="mp_helicality" weight="0"/>

<Reweight scoretype="mp_span_ang" weight="0"/>

<Reweight scoretype="mp_nonhelix" weight="0"/>

</ScoreFunction>

<ScoreFunction name="score5" weights="%%score_func_5%%" symmetric="1">

<Reweight scoretype="mp_helicality" weight="0"/>

<Reweight scoretype="mp_span_ang" weight="0"/>

<Reweight scoretype="mp_nonhelix" weight="0"/>

</ScoreFunction>

<ScoreFunction name="ref" weights="ref2015_memb" symmetric="1">

<Reweight scoretype="mp_helicality" weight="0"/>

<Reweight scoretype="mp_span_ang" weight="0"/>

</ScoreFunction>

<ScoreFunction name="refNotSymm" weights="ref2015_memb" symmetric="0">

<Reweight scoretype="mp_helicality" weight="0"/>

<Reweight scoretype="mp_span_ang" weight="0"/>

</ScoreFunction>

<ScoreFunction name="helicality" symmetric="1">

<Reweight scoretype="mp_helicality" weight="0"/>

<Reweight scoretype="mp_span_ang" weight="0"/>

</ScoreFunction>

<ScoreFunction name="helicality_notsymm" symmetric="0">

<Reweight scoretype="mp_helicality" weight="0"/>

<Reweight scoretype="mp_span_ang" weight="0"/>

</ScoreFunction>

</SCOREFXNS>

<MOVERS>

<SetupForSymmetry name="symm" definition="%%symm_file%%"/>

<SymmetricAddMembraneMover name="add_memb" membrane_core="%%membrane_core%%" steepness="%%steepness%%" span_starts_num="%%span_starts%%" span_ends_num="%%span_ends%%" span_orientations="%%span_oris%%"/>

<MembranePositionFromTopologyMover name="init_pos"/>

<FastRelax name="fast_relax" scorefxn="%%energy_function%%" task_operations="init"/>

Fragment movers

<SingleFragmentMover name="frag9" fragments="%%frags9mers%%" policy="uniform">

<MoveMap>

<Span begin="1" end="24" chi="1" bb="1"/>

</MoveMap>

</SingleFragmentMover>

<SingleFragmentMover name="frag3" fragments="%%frags3mers%%" policy="smooth">

<MoveMap>

<Span begin="1" end="24" chi="1" bb="1"/>

</MoveMap>

</SingleFragmentMover>

Fold-and-dock specific movers

<SymFoldandDockRbTrialMover name="rbtrial" rot_mag="8.0" trans_mag="3.0" rotate_anchor_to_x="1"/>

<SymFoldandDockRbTrialMover name="rbtrial_smooth" rot_mag="1.0" trans_mag="0.1" rotate_anchor_to_x="1"/>

<SymFoldandDockMoveRbJumpMover name="rbjump"/>

<SymFoldandDockSlideTrialMover name="slidetrial"/>

Random movers

<RandomMover name="early_stage_moveset" movers="frag9,rbtrial,rbjump,slidetrial" weights="1.0,0.2,1.0,0.1" repeats="1"/>

<RandomMover name="final_stage_moveset" movers="frag3,rbtrial_smooth,rbjump,slidetrial" weights="1.0,0.2,1.0,0.1" repeats="1"/>

Monte Carlo Movers

<GenericMonteCarlo name="stage1" scorefxn_name="score0" mover_name="early_stage_moveset" temperature="2.0" trials="200" recover_low="1"/>

<GenericMonteCarlo name="stage2" scorefxn_name="score1" mover_name="early_stage_moveset" temperature="2.0" trials="200" recover_low="1"/>

<GenericMonteCarlo name="stage3a" scorefxn_name="score2" mover_name="early_stage_moveset" temperature="2.0" trials="20" recover_low="1"/>

<GenericMonteCarlo name="stage3b" scorefxn_name="score5" mover_name="early_stage_moveset" temperature="2.0" trials="20" recover_low="1"/>

<GenericMonteCarlo name="stage4" scorefxn_name="score3" mover_name="final_stage_moveset" temperature="2.0" trials="400" recover_low="1"/>

Special stage 3 logic

<ParsedProtocol name="stage3_cyc">

<Add mover="stage3a"/>

<Add mover="stage3b"/>

</ParsedProtocol>

<LoopOver name="stage3" mover_name="stage3_cyc" iterations="5" drift="1"/>

Converts the centroid-level pose to fullatom for scoring

<SwitchResidueTypeSetMover name="fullatom" set="fa_standard"/>

<ExtractAsymmetricPose name="extract_asp" clear_sym_def="1"/>

<MinMover name="min_mover" scorefxn="refNotSymm" chi="1" bb="1" jump="1"/>

<PackRotamersMover name="pack" scorefxn="refNotSymm" task_operations="init,rtr"/>

<RotamerTrialsMinMover name="RTmin" scorefxn="refNotSymm" task_operations="init,rtr"/>

<DumpPdb name="dump_pdb" fname="dump.pdb" scorefxn="%%energy_function%%"/>

<SwitchChainOrder name="switch" chain_order="123"/>

<DeleteChain name="delete_mem" chain="4" />

</MOVERS>

<FILTERS>

<ScoreType name="total" scorefxn="%%energy_function%%" score_type="total_score" confidence="1" threshold="0"/>

<Sasa name="a_sasa" confidence="0" threshold="300"/>

<ResidueLipophilicity name="a_res_lipo" threshold="1000" confidence="0"/>

<SpanTopologyMatchPose name="a_span_topo" confidence="0"/>

<Ddg name="a_ddg" scorefxn="%%energy_function%%NotSymm" chain_num="2" repeats="5" extreme_value_removal="true" confidence="0" threshold="-5"/>

<PackStat name="a_pack" confidence="0" threshold="0.3"/>

<BuriedUnsatHbonds2 name="a_unsat" scorefxn="%%energy_function%%" confidence="0"/>

<ShapeComplementarity name="a_shape" confidence="0"/>

<TMsSpanMembrane name="a_tms_span" confidence="1" min_distance="25"/>

<TMsSpanMembrane name="a_tms_span_fa" confidence="0" min_distance="25"/>

<HelixHelixAngle name="a_hha_ang" angle_or_dist="angle" start_helix_1="%%span_start_1%%" end_helix_1="%%span_end_1%%" start_helix_2="%%span_start_2%%" end_helix_2="%%span_end_2%%" confidence="0"/>

<HelixHelixAngle name="a_hha_dst_vec" angle_or_dist="dist" dist_by_atom="0" start_helix_1="%%span_start_1%%" end_helix_1="%%span_end_1%%" start_helix_2="%%span_start_2%%" end_helix_2="%%span_end_2%%" confidence="0"/>

<HelixHelixAngle name="a_hha_dst_atm" angle_or_dist="dist" dist_by_atom="1" start_helix_1="%%span_start_1%%" end_helix_1="%%span_end_1%%" start_helix_2="%%span_start_2%%" end_helix_2="%%span_end_2%%" confidence="0"/>

<MembAccesResidueLipophilicity name="a_marl" confidence="0" verbose="0"/>

<ScoreType name="a_helicality" scorefxn="helicality_notsymm" score_type="mp_helicality" confidence="0" threshold="10"/>

<ScoreType name="a_helicality_symm" scorefxn="helicality" score_type="mp_helicality" confidence="0" threshold="10"/>

<MPSpanAngle name="a_angle_1" tm="1" ang_min="0" ang_max="50" confidence="0"/>

<MPSpanAngle name="a_angle_2" tm="2" ang_min="0" ang_max="50" confidence="0"/>

<BindingStrain name="a_bind" scorefxn="%%energy_function%%" jump="1" confidence="0" threshold="5"/>

<PoseInfo name="info"/>

</FILTERS>

<PROTOCOLS>

<Add mover="symm"/>

<Add mover="add_memb"/>

<Add mover="stage1"/>

<Add mover="stage2"/>

<Add mover="stage3"/>

<Add mover="stage4"/>

<Add filter="a_helicality_symm"/>

<Add filter="a_angle_1"/>

<Add filter="a_angle_2"/>

<Add mover="fullatom"/>

<Add filter="a_tms_span"/>

<Add mover="fast_relax"/>

<Add filter="total"/>

<Add filter="a_sasa"/>

<Add filter="a_span_topo"/>

<Add mover="extract_asp"/>

<Add mover="pack"/>

<Add mover="min_mover"/>

<Add mover="RTmin"/>

<Add mover="RTmin"/>

<Add filter="a_tms_span"/>

<Add filter="total"/>

<Add filter="a_sasa"/>

<Add filter="a_span_topo"/>

<Add filter="a_res_lipo"/>

<Add filter="a_pack"/>

<Add filter="a_unsat"/>

<Add filter="a_shape"/>

<Add filter="a_ddg"/>

<Add filter="a_hha_ang"/>

<Add filter="a_hha_dst_vec"/>

<Add filter="a_hha_dst_atm"/>

<Add filter="a_marl"/>

<Add filter="a_tms_span_fa"/>

<Add filter="a_helicality"/>

<Add filter="a_angle_1"/>

<Add filter="a_angle_2"/>

<Add filter="a_bind"/>

</PROTOCOLS>

<OUTPUT scorefxn="%%energy_function%%NotSymm"/>

</ROSETTASCRIPTS>
