# Supplementary figures and images for: De novo-designed transmembrane domains tune engineered receptor functions
Source: eLife. 2022 May 4;11:e75660. doi: 10.7554/eLife.75660 (PMC9068223; doi:10.7554/eLife.75660)

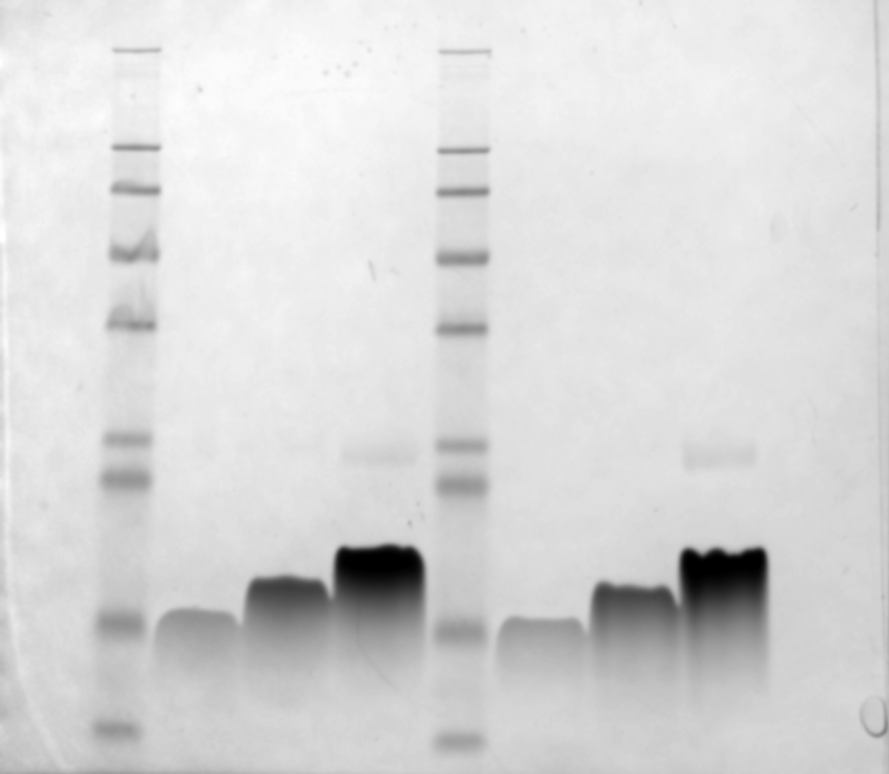

Supplement: Source data 1. [file elife-75660-data1.zip › Gels and blots for review/Figure S3/FigureS3_proMP1.1.tif]

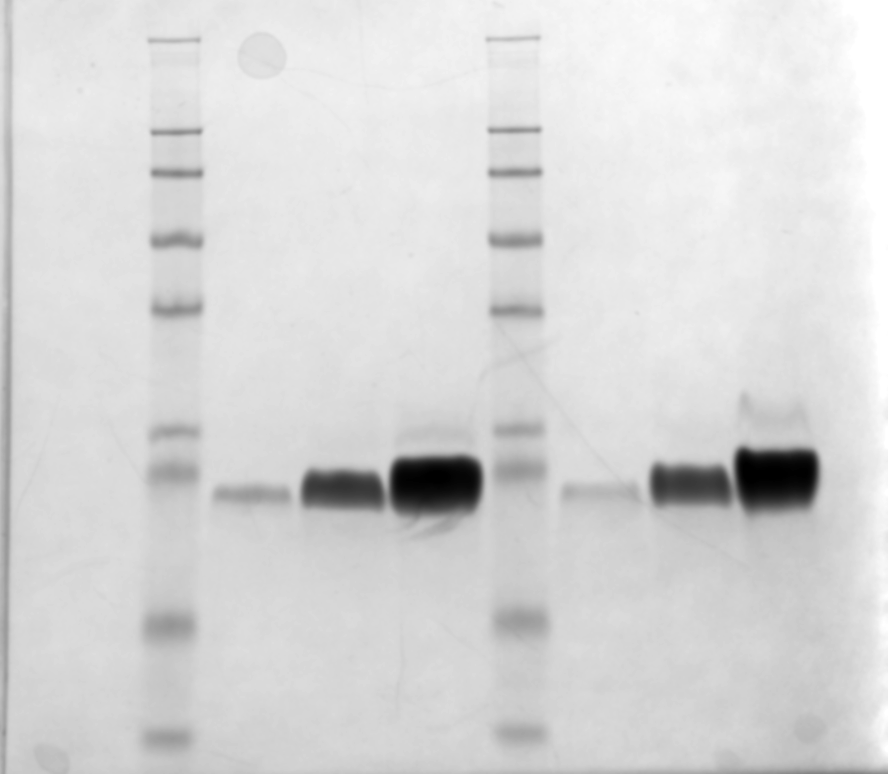

Supplement: Source data 1. [file elife-75660-data1.zip › Gels and blots for review/Figure S3/FigureS3_proMP1.3.tif]

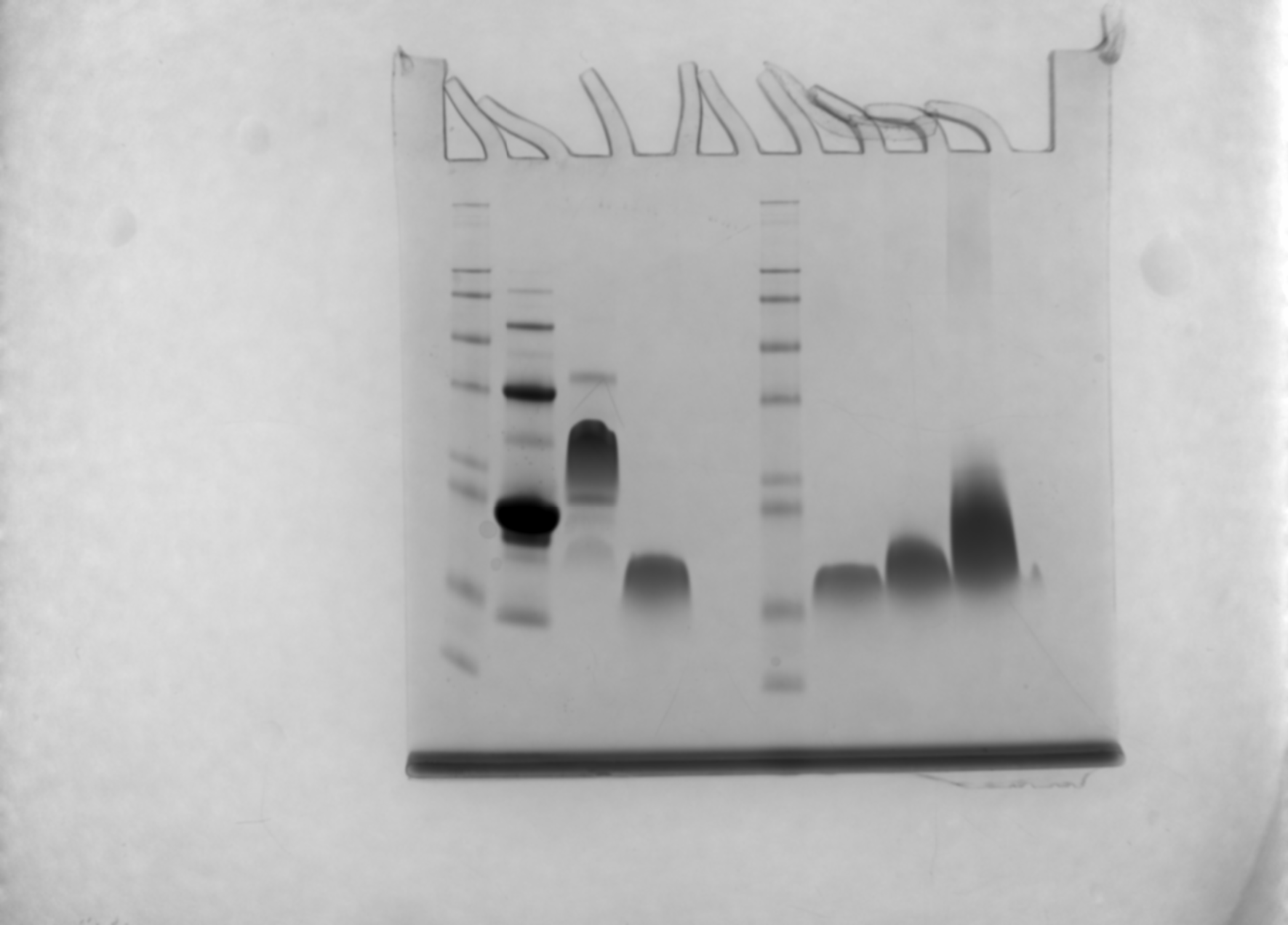

Supplement: Source data 1. [file elife-75660-data1.zip › Gels and blots for review/Figure S3/FigureS3_GpA.tif]

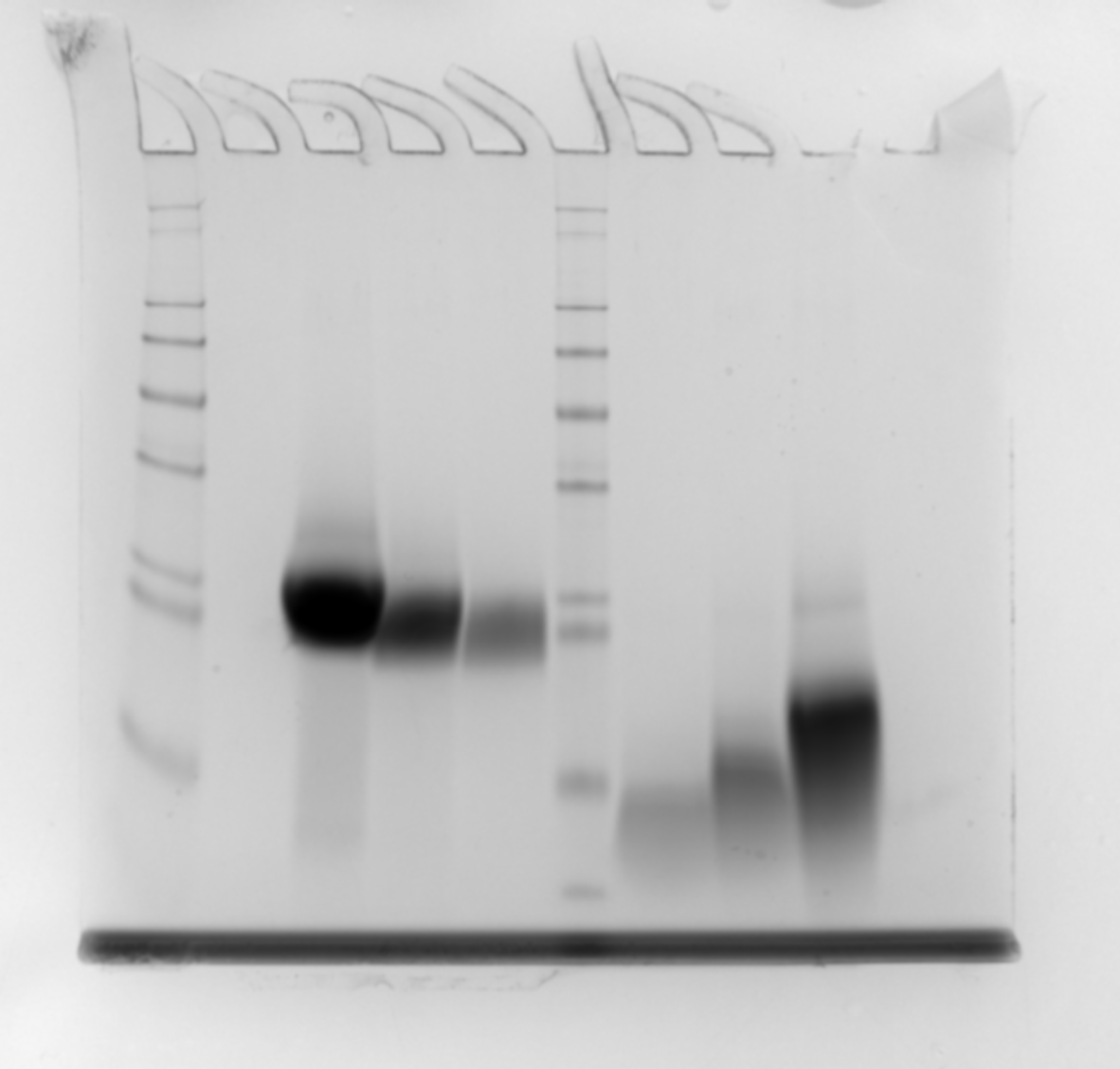

Supplement: Source data 1. [file elife-75660-data1.zip › Gels and blots for review/Figure S3/FigureS3_proMP1.12.tif]

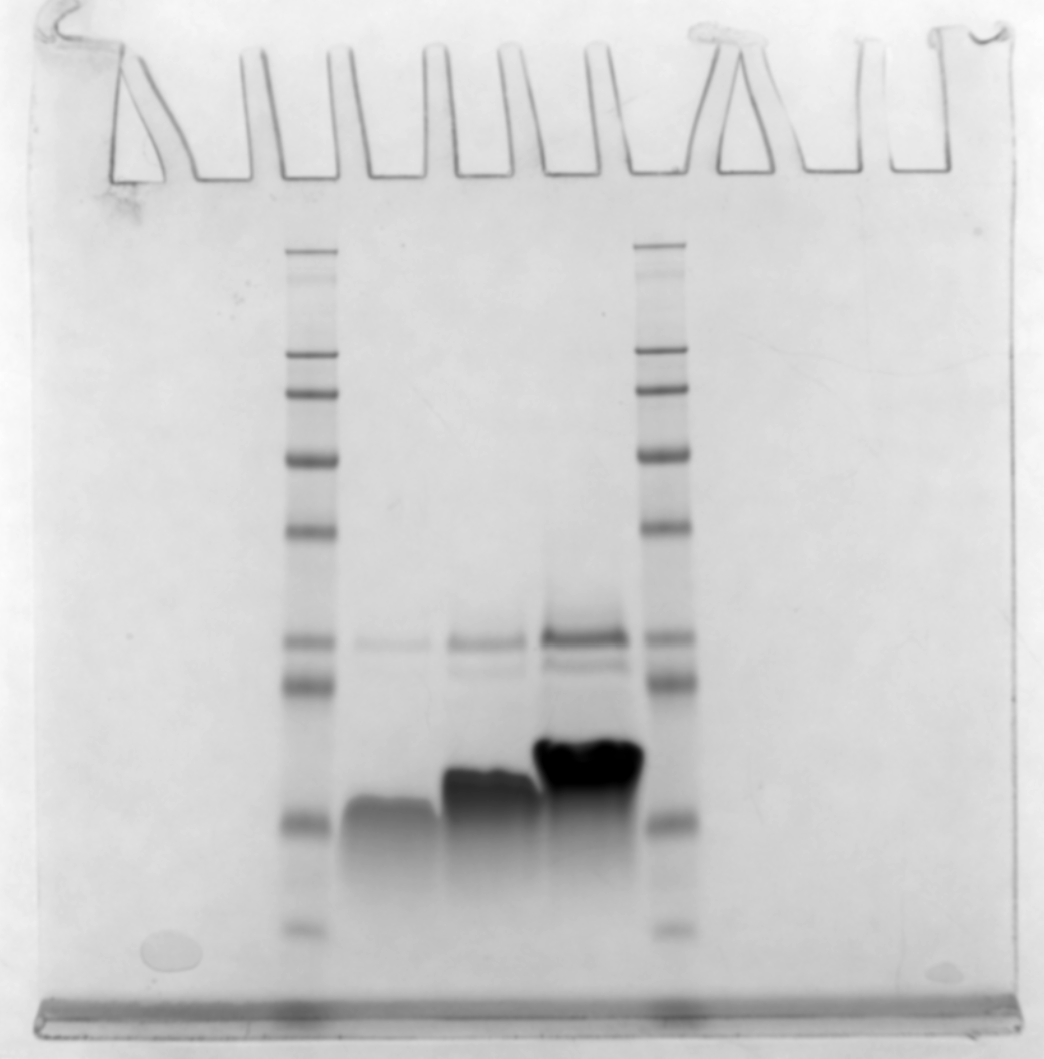

Supplement: Source data 1. [file elife-75660-data1.zip › Gels and blots for review/Figure S3/FigureS3_proMP1.9.tif]

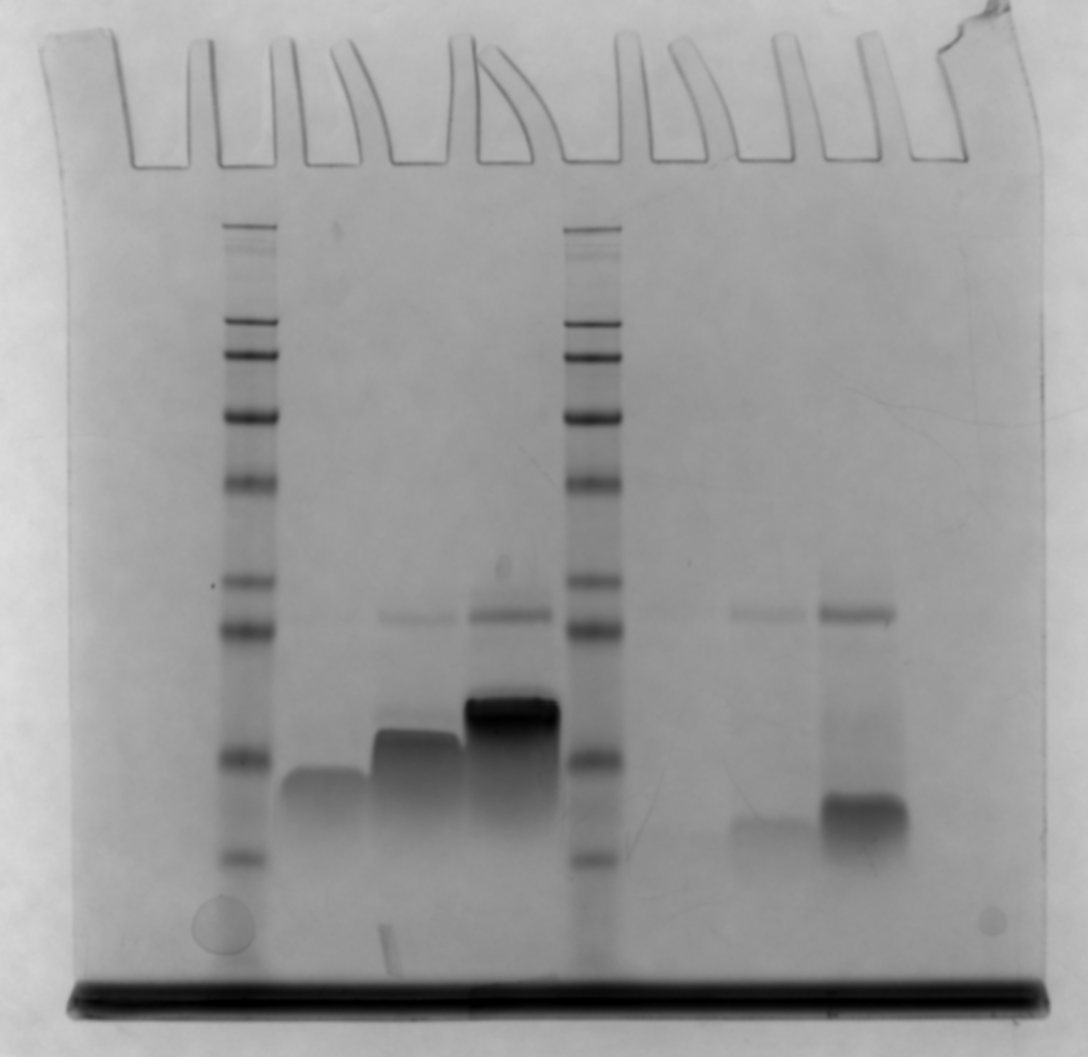

Supplement: Source data 1. [file elife-75660-data1.zip › Gels and blots for review/Figure S3/FigureS3_proMP1.8.tif]

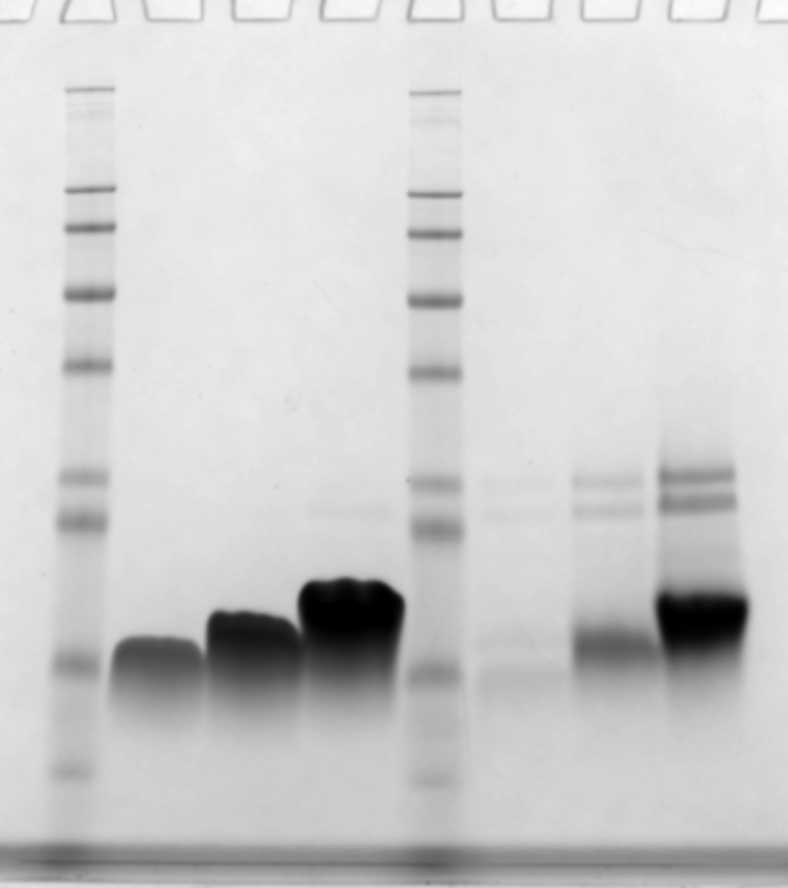

Supplement: Source data 1. [file elife-75660-data1.zip › Gels and blots for review/Figure 1/Fig1G_proMP1.5.tif]

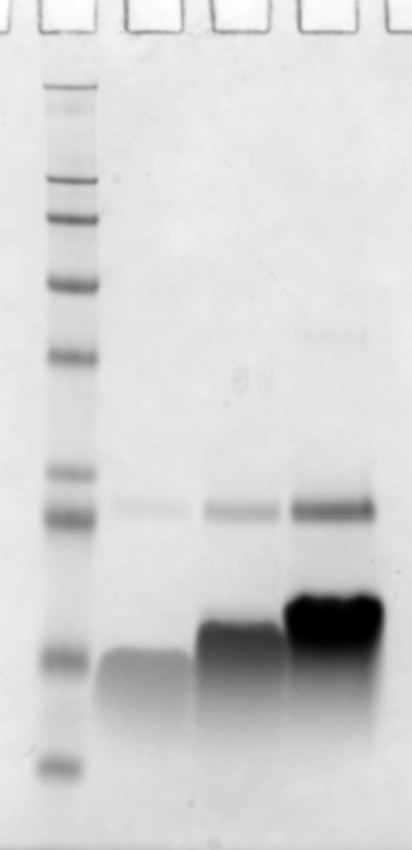

Supplement: Source data 1. [file elife-75660-data1.zip › Gels and blots for review/Figure 1/Fig1G_proMP1.6.tif]

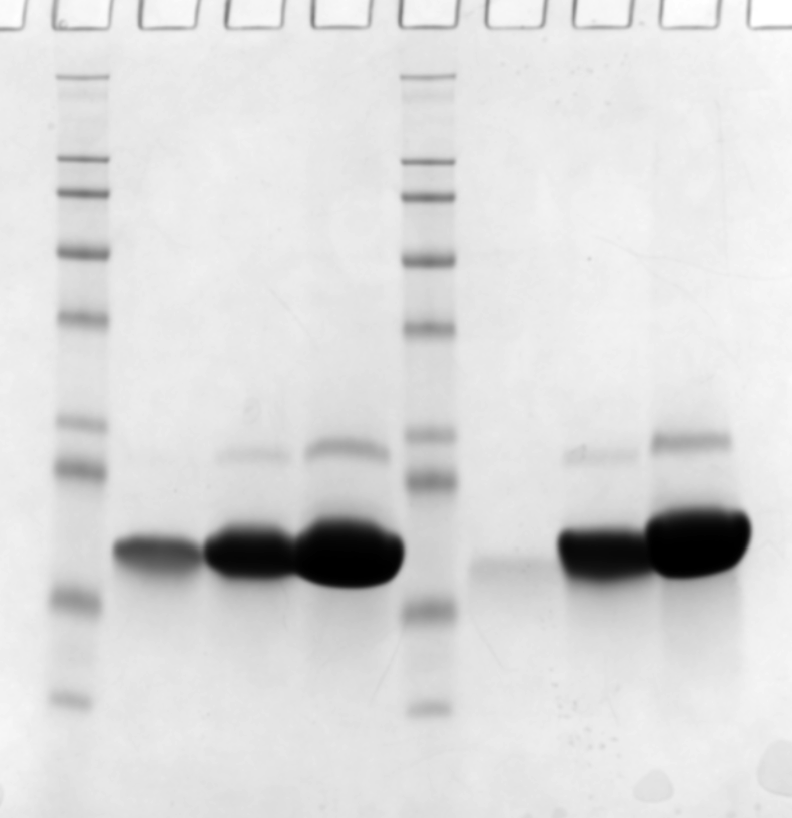

Supplement: Source data 1. [file elife-75660-data1.zip › Gels and blots for review/Figure 1/Fig1G_proMP1.2.tif]

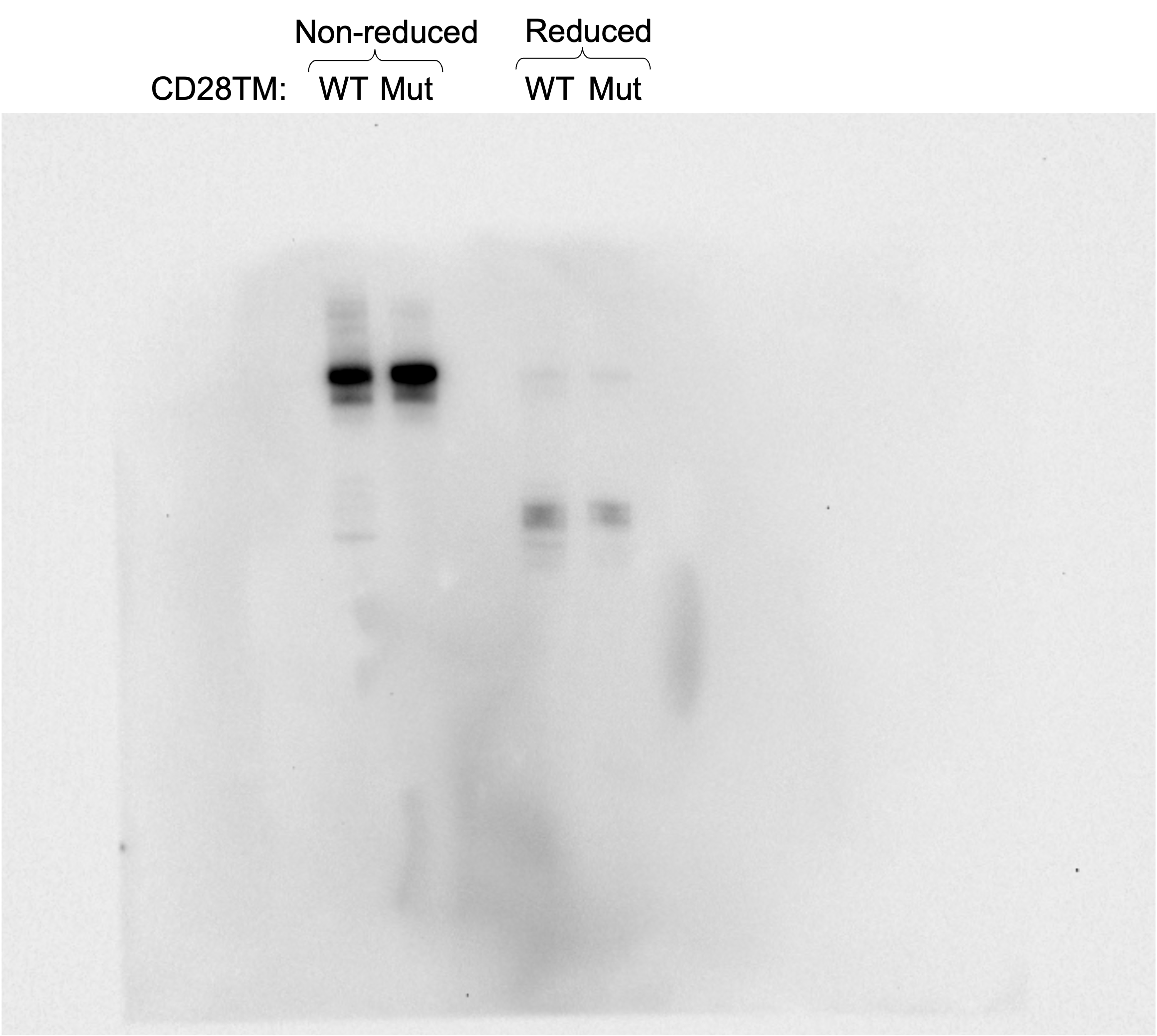

Supplement: Source data 1. [file elife-75660-data1.zip › Gels and blots for review/Figure 4/Figure 4c annotated.png]

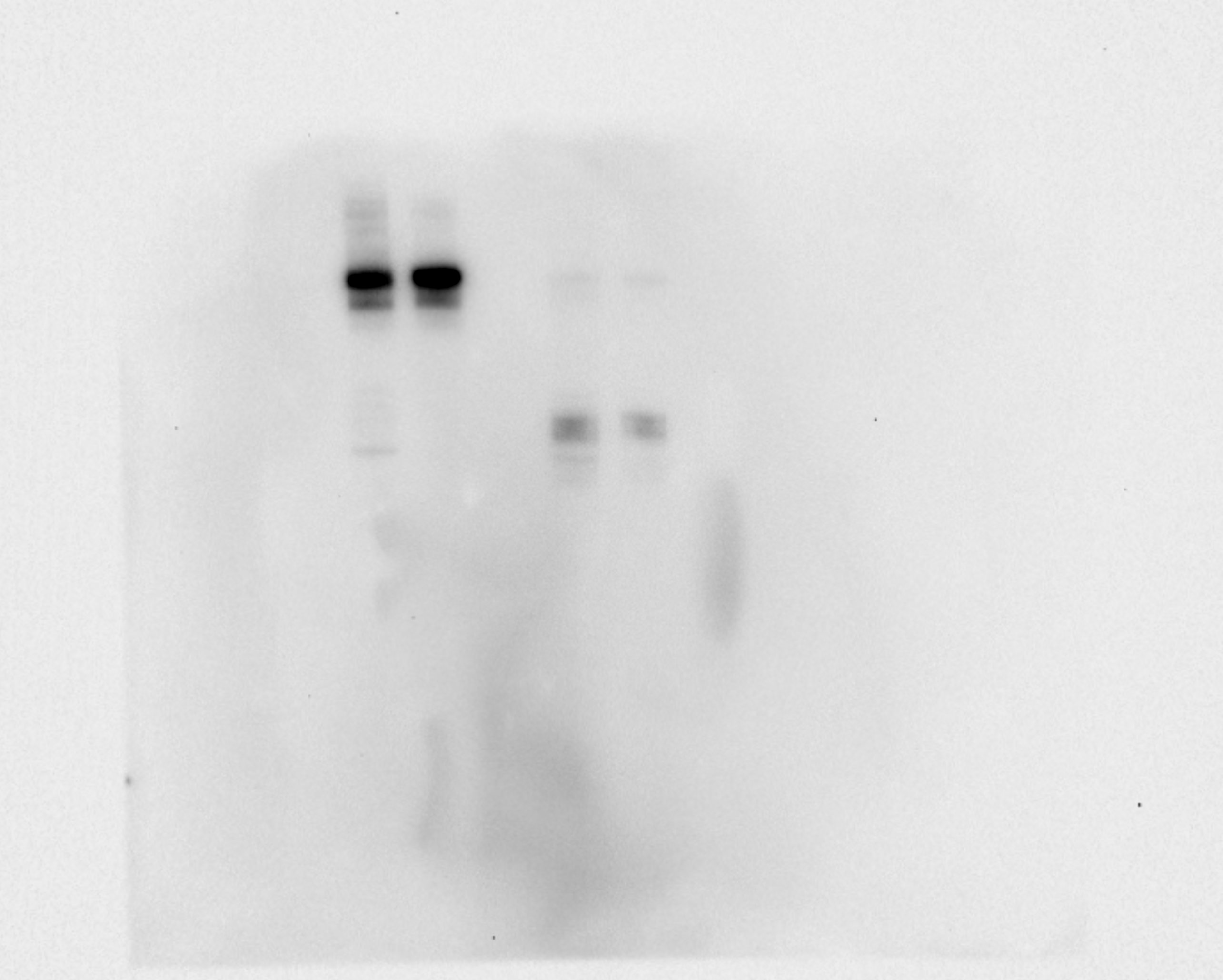

Supplement: Source data 1. [file elife-75660-data1.zip › Gels and blots for review/Figure 4/Figure 4c.png]

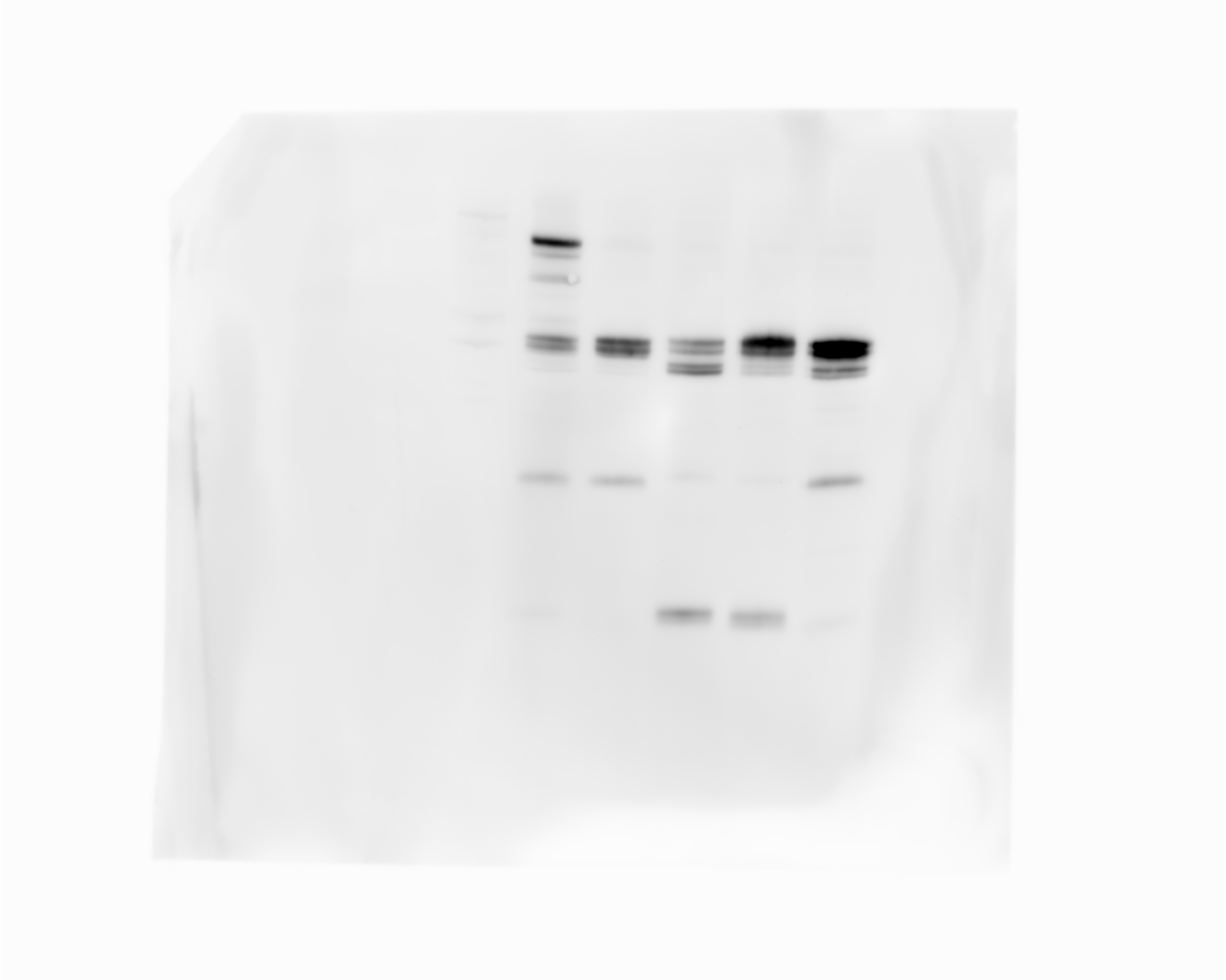

Supplement: Source data 1. [file elife-75660-data1.zip › Gels and blots for review/Figure 3/Figure 3c.png]

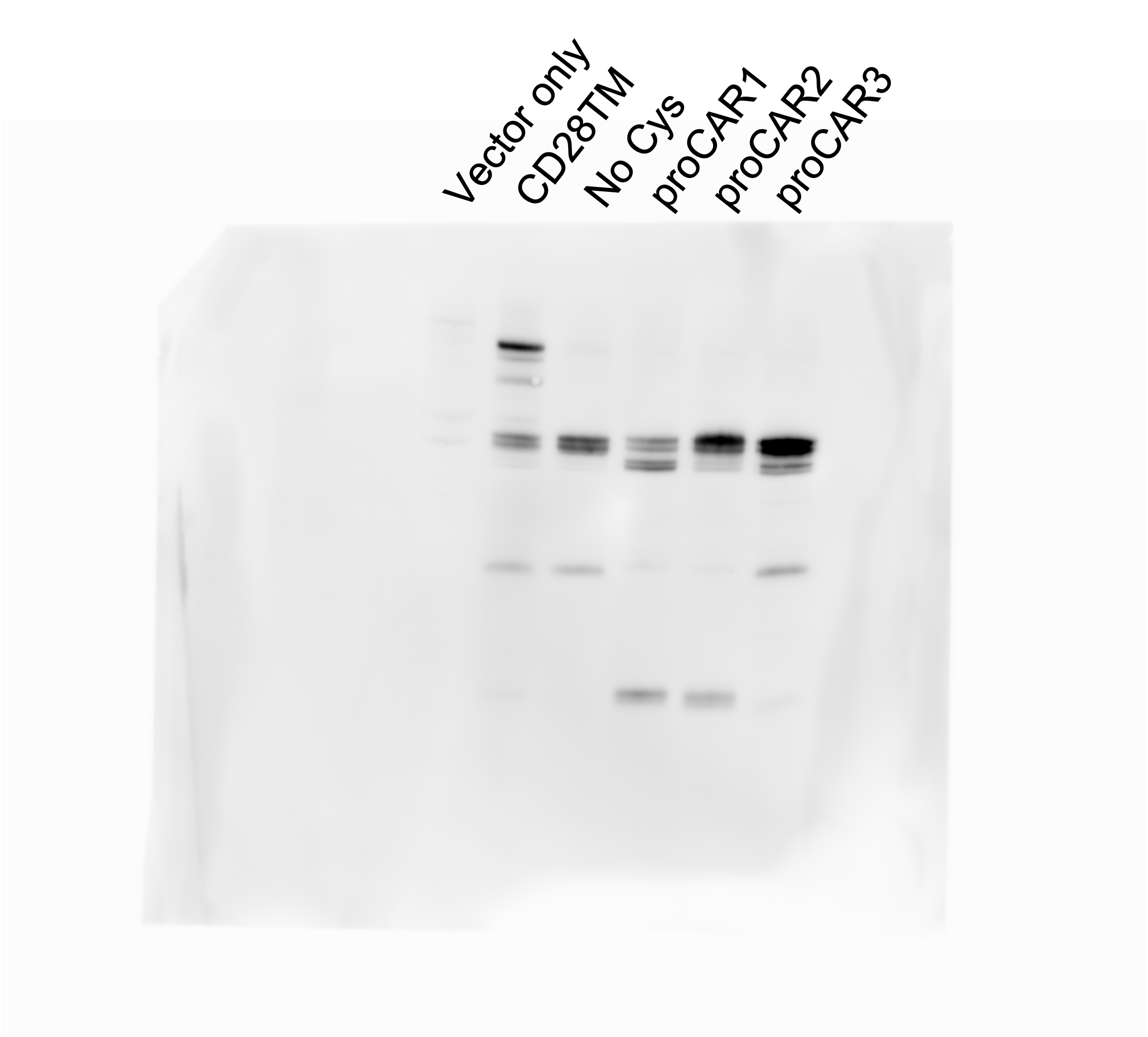

Supplement: Source data 1. [file elife-75660-data1.zip › Gels and blots for review/Figure 3/Figure 3c annotated.png]

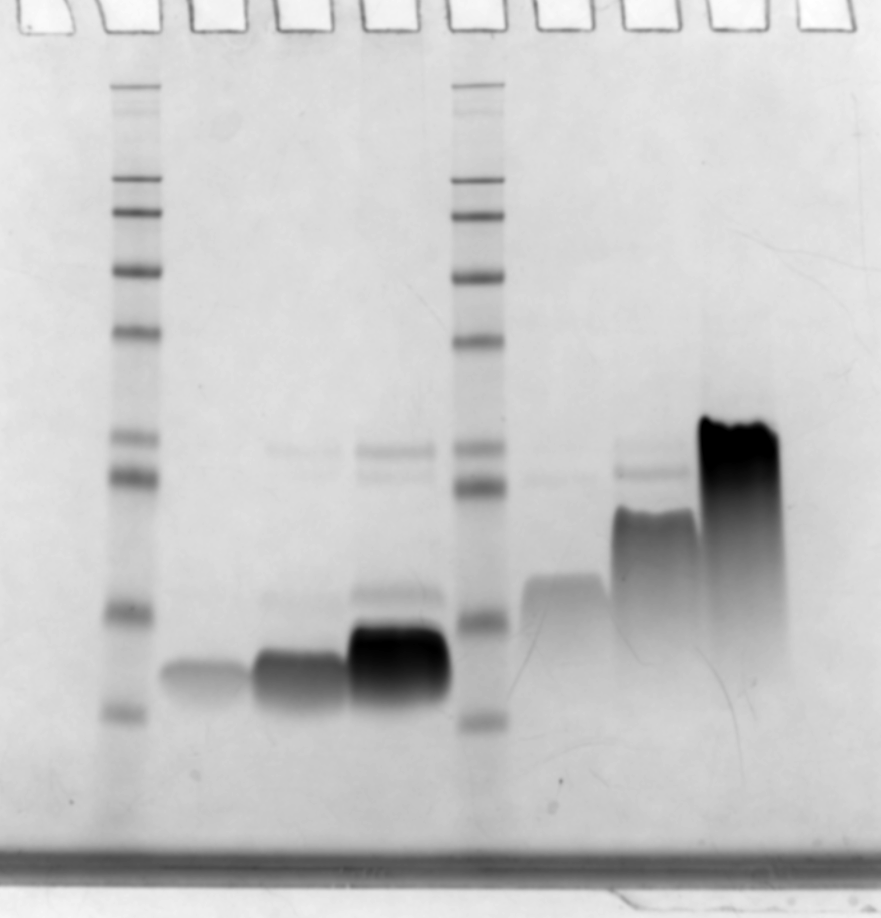

Supplement: Source data 1. [file elife-75660-data1.zip › Gels and blots for review/Figure 2/Figure 2C_proMPC3.1.tif]

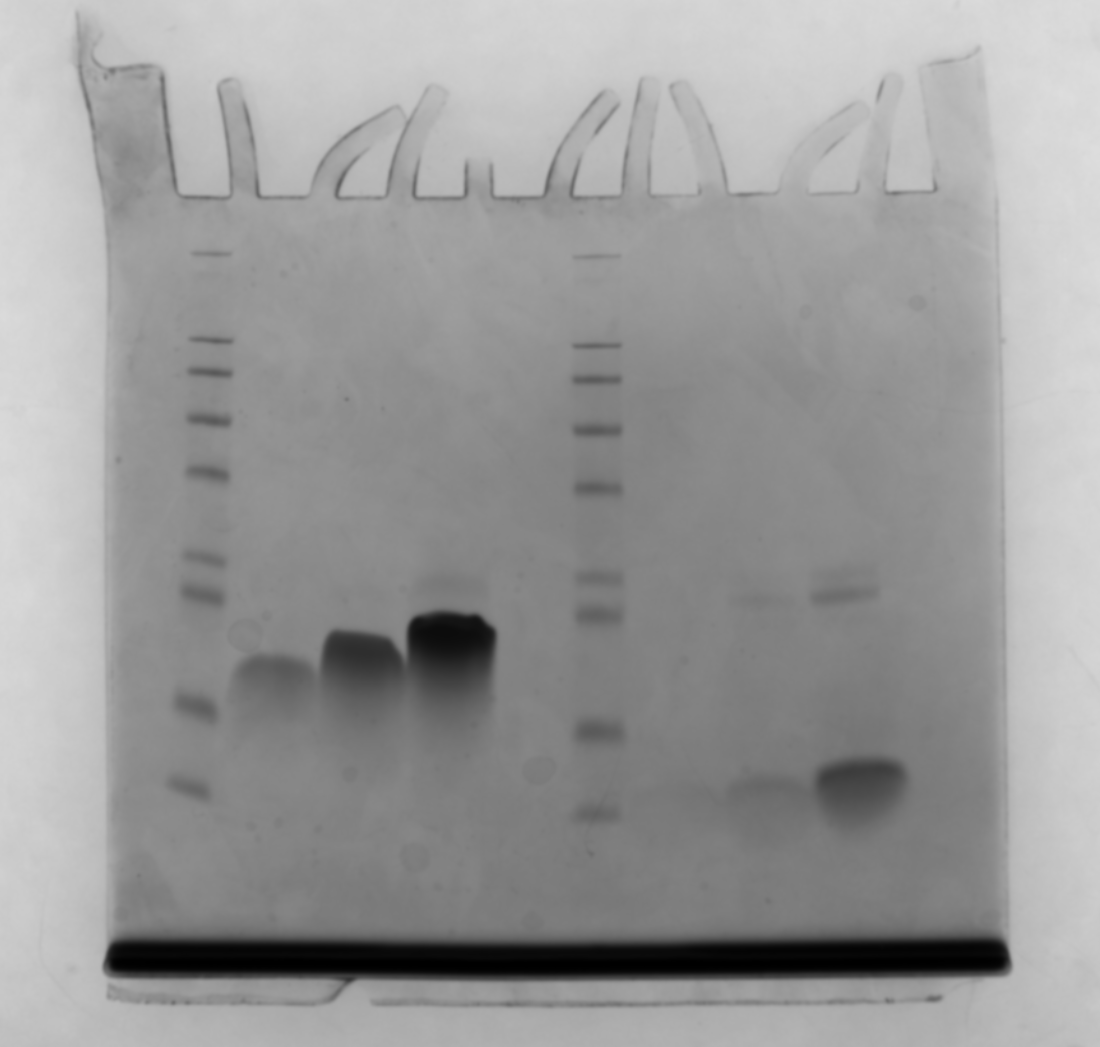

Supplement: Source data 1. [file elife-75660-data1.zip › Gels and blots for review/Figure 2/Figure2A_proMPC2.1.tif]
