# Supplementary material for: De novo-designed transmembrane domains tune engineered receptor functions
Source: eLife. 2022 May 4;11:e75660. doi: 10.7554/eLife.75660 (PMC9068223; doi:10.7554/eLife.75660)
Supplement: Transparent reporting form [file elife-75660-transrepform1.docx]

***eLife’s* transparent reporting form**

We encourage authors to provide detailed information *within their submission* to facilitate the interpretation and replication of experiments. Authors can upload supporting documentation to indicate the use of appropriate reporting guidelines for health-related research (see [EQUATOR Network](http://www.equator-network.org/%20)), life science research (see the [BioSharing Information Resource](https://biosharing.org/)), or the [ARRIVE guidelines](http://www.plosbiology.org/article/info:doi/10.1371/journal.pbio.1000412) for reporting work involving animal research. Where applicable, authors should refer to any relevant reporting standards documents in this form.

If you have any questions, please consult our Journal Policies and/or contact us: [editorial@elifesciences.org](mailto:editorial@elifesciences.org).

**Sample-size estimation**

- You should state whether an appropriate sample size was computed when the study was being designed
- You should state the statistical method of sample size computation and any required assumptions
- If no explicit power analysis was used, you should describe how you decided what sample (replicate) size (number) to use

Please outline where this information can be found within the submission (e.g., sections or figure legends), or explain why this information doesn’t apply to your submission:

Our animal ethics at The Walter and Eliza Hall Institute (Ethics #2020.030) has tight regulations regarding reducing animal numbers where possible. All our mouse experiments were performed on mice in groups of 5-6 as per our AEC approvals, as described in the methods.

**Replicates**

- You should report how often each experiment was performed
- You should include a definition of biological versus technical replication
- The data obtained should be provided and sufficient information should be provided to indicate the number of independent biological and/or technical replicates
- If you encountered any outliers, you should describe how these were handled
- Criteria for exclusion/inclusion of data should be clearly stated
- High-throughput sequence data should be uploaded before submission, with a private link for reviewers provided (these are available from both GEO and ArrayExpress)

Please outline where this information can be found within the submission (e.g., sections or figure legends), or explain why this information doesn’t apply to your submission:

All biological replicates for each experiment are defined in the figure’s corresponding figure legend. Information regarding technical replicates is stated in the methods under the appropriate experimental techniques. All data points for each experiment are shown in the figures with no outliers/data points excluded.

**Statistical reporting**

- Statistical analysis methods should be described and justified
- Raw data should be presented in figures whenever informative to do so (typically when N per group is less than 10)
- For each experiment, you should identify the statistical tests used, exact values of N, definitions of center, methods of multiple test correction, and dispersion and precision measures (e.g., mean, median, SD, SEM, confidence intervals; and, for the major substantive results, a measure of effect size (e.g., Pearson's r, Cohen's d)
- Report exact p-values wherever possible alongside the summary statistics and 95% confidence intervals. These should be reported for all key questions and not only when the p-value is less than 0.05.

Please outline where this information can be found within the submission (e.g., sections or figure legends), or explain why this information doesn’t apply to your submission:

Explanations of data presentation (e.g. N, mean and SEM) and statistical tests are included in the figure legends. Unless all data in the experiment was shown to be normally distributed, non-parametric tests were always performed and the specific test used is always stated in the corresponding figure legend. Exact p-values are stated for all statistical tests regardless of their value ranges or interpretation.

(For large datasets, or papers with a very large number of statistical tests, you may upload a single table file with tests, Ns, etc., with reference to sections in the manuscript.)

**Group allocation**

- Indicate how samples were allocated into experimental groups (in the case of clinical studies, please specify allocation to treatment method); if randomization was used, please also state if restricted randomization was applied
- Indicate if masking was used during group allocation, data collection and/or data analysis

Please outline where this information can be found within the submission (e.g., sections or figure legends), or explain why this information doesn’t apply to your submission:

For all mouse experiments, mice were allocated to treatment groups at random. Tumours were not yet detectable at the time of CAR-T cell treatment, so tumour size was not taken into consideration. Further, all mice were weighed prior to treatment and all groups had a similar average total body weight. No masking was performed for the mouse experiments, however final tumour weight was recorded for all mouse experiments and no difference in final tumour weight was observed between groups, supporting an absence of experimenter bias.

**Additional data files (“source data”)**

- We encourage you to upload relevant additional data files, such as numerical data that are represented as a graph in a figure, or as a summary table
- Where provided, these should be in the most useful format, and they can be uploaded as “Source data” files linked to a main figure or table
- Include model definition files including the full list of parameters used
- Include code used for data analysis (e.g., R, MatLab)
- Avoid stating that data files are “available upon request”

Please indicate the figures or tables for which source data files have been provided:

Source code for the de novo design strategy is provided in Supplementary File 1. Source files for all gels and blots are provided separately. Structure coordinates and validation reports are available from the Protein Data Bank under accession codes 6W9Y, 6W9Z and 6WA0.
